# Isolated congenital hair tuft at an unusual location

**DOI:** 10.1016/j.jdcr.2025.04.042

**Published:** 2025-06-02

**Authors:** Maira A. Bhatty, Paul X. Benedetto, Todd Stultz, Allison Vidimos

**Affiliations:** a Case Western Reserve University School of Medicine, Cleveland, Ohio; b Department of Dermatology, Cleveland Clinic, Coral Springs, Florida; c Department of Diagnostic Radiology, Cleveland Clinic, Cleveland, Ohio; d Department of Dermatology, Cleveland Clinic, Cleveland, Ohio

**Keywords:** faun tail nevus, spina bifida occulta, thoracic hypertrichosis

## History

**Fig 1:**
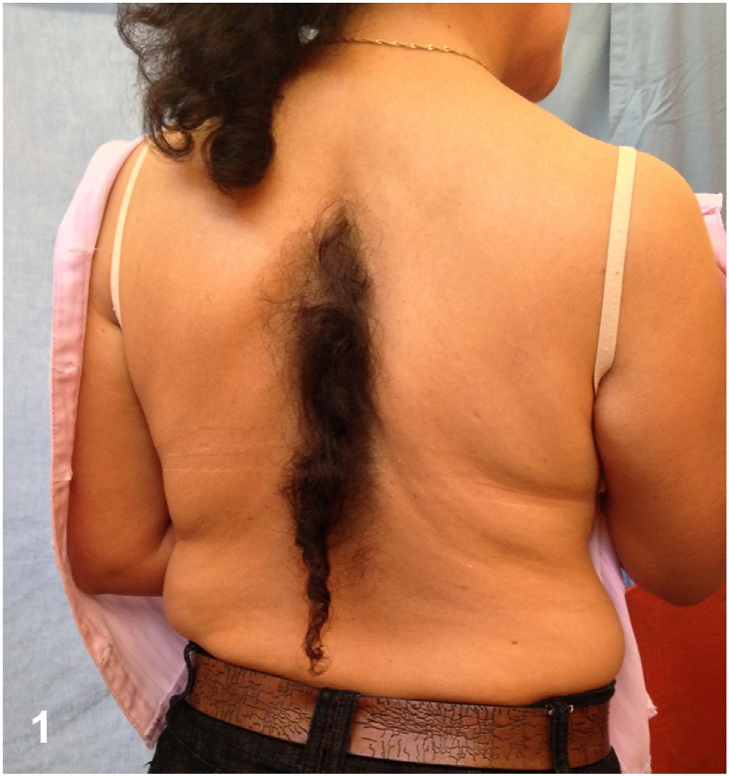

A 43-year-old Hispanic woman with an unremarkable medical and family history underwent a routine health screening on an international medical service trip in Honduras. Auscultation of her posterior chest revealed an 8-inch tuft of hair overlying the central thoracic spine (Fig 1). The patient noted that the hair on her spine had been present since birth but had not caused her any issues, so she had never sought medical evaluation. There was no associated pitting, discoloration, depression, or atrophy underlying the terminal hair shafts. Review of systems and complete neurological exam were unremarkable.

**Question 1: What is the most likely diagnosis in this patient?**

A. Hirsutism
B. Anorexia nervosa
C. Congenital hypertrichosis lanuginosa
D. Localized hypertrichosis
E. Porphyria cutanea tarda

**Answers:**

A. Hirsutism – Incorrect. Hirsutism presents with androgen-dependent terminal hair growth in women, and it may be associated with other signs of androgen excess like acne, androgenetic alopecia, and menstrual irregularities.
B. Anorexia nervosa – Incorrect. Anorexia nervosa presents with generalized lanugo-like hair instead of a single tuft of terminal hair.
C. Congenital hypertrichosis lanuginosa – Incorrect. Congenital hypertrichosis lanuginosa is characterized by long, fine, silvery-gray to blond lanugo hair that covers the entire body, except for the palms, soles, and mucous membranes.
D. Localized hypertrichosis – Correct. Faun tail nevus, or localized hypertrichosis, is a rare congenital anomaly characterized by a triangular patch of hair, usually on the lumbosacral spine. Location over the thoracic spine is very rare. The hair can be either dark or light in color, and the texture is usually silky, although it can vary.^1^
E. Porphyria cutanea tarda – Incorrect. Porphyria cutanea tarda presents with hypertrichosis on sun-exposed areas, especially on the lateral face. In addition, other symptoms of porphyria would be expected including a blistering photosensitive eruption.

**Question 2: What is the most common association with this condition?**

A. Spinal cord malformations
B. Dental anomalies
C. Photosensitive rash
D. Dysmorphic facies
E. Thick bushy eyebrows

**Answers:**

A. Spinal cord malformations – Correct. Localized hypertrichosis is a cutaneous marker for 40% to 50% of cases of spinal dysraphism.^2^ Skin and neurological defects may arise together because of their shared ectodermal origin. Neuroectoderm separates from epithelial ectoderm in the third to fifth week of a fetus’ life, and incomplete cleavage can result in a defect involving the skin, spinal cord, and/or vertebrae.^2^^,^^3^ Left untreated, the vertebral column can grow disproportionately to the spinal cord, leading to neurologic deficits. The spinal defects most commonly associated with localized hypertrichosis are spina bifida, intraspinal lipomas, dermal sinuses, lipomeningomyeloceles, diastematomyelia, tethered cord, hemivertebra, and incomplete vertebra.^3^
B. Dental anomalies – Incorrect. Dental anomalies are occasionally associated with congenital hypertrichosis lanuginosa.
C. Photosensitive rash – Incorrect. A photosensitive rash is commonly observed in porphyria cutanea tarda.
D. Dysmorphic facies – Incorrect. Dysmorphic facies can be seen in hypertrichosis universalis congenita, or Ambras syndrome. It can also be accompanied by supernumerary nipples and dental anomalies.
E. Thick bushy eyebrows – Incorrect. Thick, bushy eyebrows and an “inverted fir tree” pattern of hair growth are associated with prepubertal hypertrichosis.

**Question 3: What is the next best step in management?**

A. Observation
B. Magnetic resonance imaging
C. Genetic testing
D. Surgical excision
E. Chemical depilatories

**Answers:**

A. Observation – Incorrect. All patients with localized lumbosacral hypertrichosis should undergo neurological examination and imaging due to the risk of underlying spinal cord or bony abnormalities.^2^ These underlying defects can manifest as debilitating sequelae with advancing age if left untreated.^4^
B. Magnetic resonance imaging – Correct. Localized lumbosacral hypertrichosis may be the first presenting sign of an underlying spinal cord defect. Therefore, all patients with localized hypertrichosis, including those without neurological deficits, require a thorough neurological examination and magnetic resonance imaging of the spine.^2^ Early diagnosis is critical since these defects may be surgically correctable before the onset of permanent neurologic damage.^1^^,^^2^ Our patient was an otherwise healthy adult, and given the unique circumstances and limited resources, a shared decision was made to forgo imaging.
C. Genetic testing – Incorrect. Currently, there is no established genetic test specifically for diagnosing a faun tail nevus or localized hypertrichosis.
D. Surgical excision – Incorrect. Studies have shown that the most effective and scar-free treatment for permanent hair reduction in localized hypertrichosis is intense pulsed light and laser epilation, specifically Alexandrite, diode, ruby, and Nd:YAG lasers.^4^^,^^5^
E. Chemical depilatories – Incorrect. Although chemical depilatories offer a quick and easy solution for temporary hair removal, they can cause irritation, are associated with allergic contact dermatitis, and are only suitable for small areas.^4^ The most effective treatment for permanent hair reduction in localized hypertrichosis is intense pulsed light and laser epilation, as discussed above.^4^^,^^5^

## Conflicts of interest

None disclosed.
